# Validated Impacts of N6-Methyladenosine Methylated mRNAs on Apoptosis and Angiogenesis in Myocardial Infarction Based on MeRIP-Seq Analysis

**DOI:** 10.3389/fmolb.2021.789923

**Published:** 2022-01-28

**Authors:** Yingjie Zhang, Wenjie Hua, Yini Dang, Yihui Cheng, Jiayue Wang, Xiu Zhang, Meiling Teng, Shenrui Wang, Min Zhang, Zihao Kong, Xiao Lu, Yu Zheng

**Affiliations:** ^1^ Department of Rehabilitation Medicine, The First Affiliated Hospital of Nanjing Medical University, Nanjing, China; ^2^ Department of Gastroenterology, The First Affiliated Hospital of Nanjing Medical University, Nanjing, China

**Keywords:** myocardial infarction, m^6^A methylation, mRNA, angiogenesis, apoptosis

## Abstract

**Objectives**: N6-methyladenosine (m^6^A) is hypothesized to play a role in the regulation of pathogenesis of myocardial infarction (MI). This study was designed to compare m^6^A-tagged transcript profiles to identify mRNA-specific changes on pathophysiological variations after MI.

**Methods**: N6-methyladenosine methylated RNA immunoprecipitation sequencing (MeRIP-seq) and RNA sequencing (RNA-seq) were interacted to select m^6^A-modified mRNAs with samples collected from sham operated and MI rat models. m^6^A methylation regulated mRNAs were interacted with apoptosis/angiogenesis related genes in GeneCards. Afterwards, MeRIP-quantitative real-time PCR (MeRIP-qRT-PCR) was performed to measure m^6^A methylation level of hub mRNAs. m^6^A methylation variation was tested under different oxygen concentration or hypoxic duration in H9c2 cells and HUVECs. In addition, Western blot and qRT-PCR were employed to detect expression of hub mRNAs and relevant protein level. Flow cytometry and Tunel assay were conducted to assess apoptotic level. CCK-8, EdU, and tube formation assay were performed to measure cell proliferation and tube formation ability.

**Results**: Upregulation of Mettl3 was firstly observed *in vivo* and *in vitro*, followed by upregulation of m^6^A methylation level. A total of 567 significantly changed m^6^A methylation peaks were identified, including 276 upregulated and 291 downregulated peaks. A total of 576 mRNAs were upregulated and 78 were downregulated. According to combined analysis of MeRIP-seq and RNA-seq, we identified 26 significantly hypermethylated and downregulated mRNAs. Based on qRT-PCR and interactive analysis, Hadh, Kcnn1, and Tet1 were preliminarily identified as hub mRNAs associated with apoptosis/angiogenesis. MeRIP-qRT-PCR assay confirmed the results from MeRIP-seq. With the inhibition of Mettl3 in H9c2 cells and HUVECs, downregulated m^6^A methylation level of total RNA and upregulated expression of hub mRNAs were observed. Increased m^6^A level was verified in the gradient context in terms of prolonged hypoxic duration and decreased oxygen concentration. Under simulated hypoxia, roles of Kcnn1 and Tet1 in angiogenesis and Hadh, Tet1, and Kcnn1 in apoptosis were further confirmed with our validation experiments.

**Conclusion**: Roles of m^6^A-modified mRNA transcripts in the context of MI were preliminarily verified. In the context of m^6^A methylation, three hub mRNAs were validated to impact the process of apoptosis/angiogenesis. Our study provided theoretical basis and innovative targets for treatment of MI and paved the way for future investigations aiming at exploring upstream epigenetic mechanisms of pathogenesis after MI.

## 1 Introduction

Myocardial infarction (MI), due to the reduction or interruption of the blood supply of the coronary artery, always results in ischemia of the corresponding myocardium leading to myocardial necrosis (Saleh and Ambrose, 2018). It is characterized by an elevated ST-segment in the electrocardiogram, and is one of the most common causes of death worldwide (Lu et al., 2015). At present, the reperfusion therapy to restore the blood circulation of the heart has become a common method for the treatment of MI, however the subsequent reperfusion injury would impair endothelial function and aggravate myocardial cell death (Thygesen et al., 2007; Puymirat et al., 2019). Upon these concerns, exploration of strategies on compensating myocardial cell regeneration and death after MI becomes the most warranted task in this research field. In addition, understanding the microscopic regulations in MI might be essential to reveal the pathophysiological mechanisms behind MI and might shed light on uncovering novel therapies for the treatment of MI.

Robust studies have explored the mechanisms of myocardial cell regeneration and death after MI. Integrin-linked kinase (ILK) has been reported to be an important factor regulating apoptosis and angiogenesis. In hypoxic condition, upregulation of ILK increased phosphorylation of protein kinase B and mammalian target of rapamycin, resulting in enhanced mesenchymal stem cells (MSCs) survival and vascular endothelial growth factors expression level. In addition, transplantation of MSCs rich in ILK could further improve angiogenesis at 3 weeks (Zeng et al., 2017). Exosomes derived from TIMP2-modified MSCs significantly increased the expression of antiapoptotic bcl-2, followed by decreased proapoptotic Bax and pro-caspase9 level, and finally attenuated apoptosis in MI injury via Akt/Sfrp2 pathway *in vivo* (Ni et al., 2019). However, the upstream regulation of these pathways has not been well documented. Recent studies on epigenetic regulation have revealed the relationships between epigenetic modifications and cardiovascular diseases. Epigenetics, including the reversible modification of DNA and protein, were proven to independently regulate gene expression of DNA and protein. It was not until recently that RNA modification was believed to be the third layer of epigenetics, regulating RNA processing and metabolism. There has been uncovered with more than 100 modifications in RNAs, including 5-methylcytosine (m^5^C), N6-methyladenosine (m^6^A), and N1-methyladenosine (m^1^A). Among which, m^6^A methylation was demonstrated to be the common and abundant internal modification of eukaryotic messenger RNA (mRNA) (Meyer et al., 2012). It is a dynamic reversible process regulated by methyltransferases (writers), demethylases (erasers), and binding proteins (readers). ALKBH5 was responsible for reducing m^6^A methylation and ALKBH5 (one of the demethylases) knockout mice exhibited decreased cardiac regenerative ability and cardiac function after neonatal apex resection (Han et al., 2021). The expression of METTL3 was increased in cardiac fibrotic tissue with chronic myocardial infarction. It promoted proliferation of cardiac fibroblasts, fibroblast-to-myofibroblast transition, and collagens accumulation, while silence of METTL3 (one of the methyltransferases) alleviated cardiac fibrosis in MI mice (Li et al., 2021). These findings emphasized the importance of m^6^A methylation in individual mRNAs and provided new insights into therapeutic strategies. Nonetheless, there is limited knowledge of the whole picture of m^6^A modification on mRNAs after MI and how performance of m^6^A methylated mRNAs on the downstream functional phenotypes.

Upon the above concerns, we aimed to systematically compare the m^6^A-tagged transcript profiles of heart tissue from rat MI models with those from sham operated (SO) rats to identify gene-specific changes in mRNA methylation and contribute to the development of MI. Specifically, we first observed the change of methyltransferases *in vivo* and *vitro* followed by the verification of m^6^A methylation in the gradient context of prolonged hypoxic duration and decreased oxygen concentration. We then identified m^6^A methylation regulated mRNAs between the SO group and the MI group by combined analysis of N6-methyladenosine methylated RNA immunoprecipitation sequencing (MeRIP-seq) and RNA sequencing (RNA-seq), then top 10 hypermethylated and downregulated mRNAs were selected and validated in rats through quantitative real-time reverse transcription-polymerase chain reaction (qRT-PCR). Afterward, we interacted the hub mRNAs with genes related to apoptosis and angiogenesis in open-source datasets. We finally validated the role of methyltransferase in the regulation of m^6^A methylation and selected hub mRNAs in angiogenesis and apoptosis. With the exploration of potential roles for the m^6^A modified mRNA transcripts in the physiological and pathological mechanisms underlying MI, a theoretical basis and innovative targets could be identified for the treatment of MI.

## 2 Materials and Methods

### 2.1 Models

#### 2.1.1 Animal MI Model

Male Sprague-Dawley (SD) rats aged 10 weeks and weighing 250–300 g were collected from Beijing Vital River Laboratory Animal Technology Co. Ltd. The current study was carried out in accordance with the guidelines of the Chinese Council on Animal Protection and approved by the Institutional Animal Care and Use Committee of Nanjing Medical University (reference number of 10091). SD rats were randomly divided into the sham operated (SO) group and the myocardial infarction (MI) group. Ligation of left anterior descending coronary artery was conducted in the MI group and myocardial ischemia was confirmed with elevated ST-segment in electrocardiogram. However, string went through the corresponding myocardial region without ligation in the SO group. Finally, heart samples were collected from the infarction area in the MI group and the corresponding region in the SO group. Heart samples were refrigerated at −80°C for further detection.

#### 2.1.2 Cell Hypoxic Model

H9c2 cells were cultured in Dulbecco’s modified eagle medium (Gibico, Waltham, CA) supplemented with 10% fetal bovine serum (Gibico, Waltham, CA) and 1% of penicillin/streptomycin at 37°C in an incubator with 95% humid air and 5% CO_2_ (Wang M. et al., 2020). Human umbilical vein endothelial cells (HUVECs) were maintained in endothelial cell growth medium-2 bullet kit (Lonza, Basel, BS, CH) at 37°C in an incubator with 95% humid air and 5% CO_2_ (Chen et al., 2020). To simulate myocardial ischemia, H9c2 cells and HUVECs were managed with hypoxia. Specifically, they were placed in a hypoxic incubator containing 94% N_2_, 5% CO_2_, and 1% O_2_ for 24 h. Meanwhile, the control group was maintained in a normal atmosphere of 95% air and 5% CO_2_ at 37°C (Zhu et al., 2021). Afterward, H9c2 cells and HUVECs were cultured in consistent 1% O_2_, 2% O_2_, and 5% O_2_ hypoxic condition for 24 h to explore the impact of different concentrations of oxygen on m^6^A methylation level. These cells were also cultured with 1% oxygen concentration for 12, 24, and 48 h to explore the impact of different hypoxic duration on m^6^A methylation level. H9c2 cells and HUVECs were then treated with 25 μM Mettl3 inhibitor (STM2457) for 24 h to explore the role of Mettl3 on m^6^A methylation level (Yankova et al., 2021).

### 2.2 MeRIP-Seq and Bioinformatic Analysis

**FIGURE 1:**
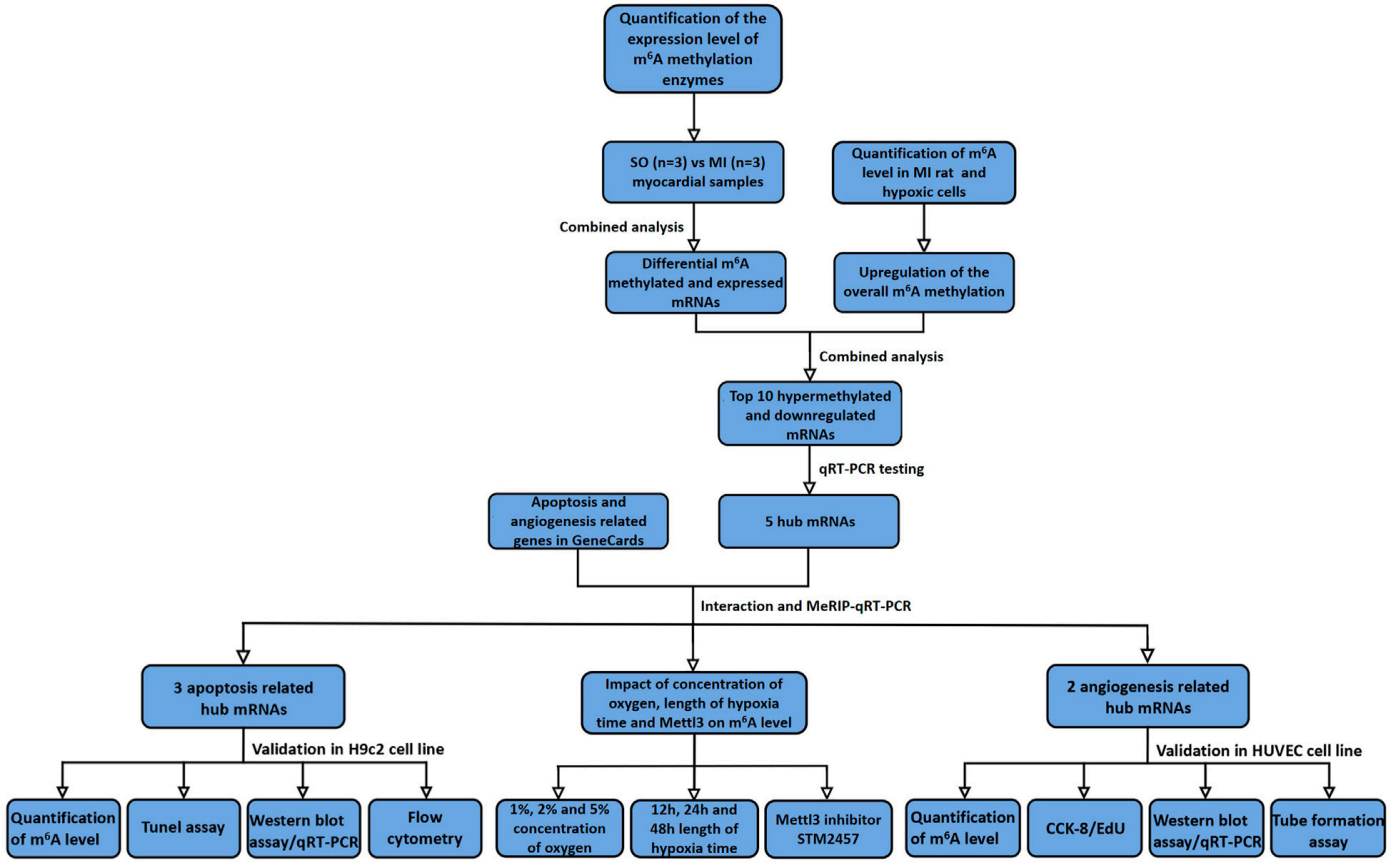
Flowchart of study procedure. SO, sham operated; MI, myocardial infarction; qRT-PCR, quantitative reverse transcription polymerase chain reaction; MeRIP-qRT-PCR, MeRIP-quantitative real-time PCR; CCK-8, cell counting kit-8; EdU, 5-ethynyl-20-deoxyuridine.

The detailed procedure of MeRIP-Seq analysis and validation of newly discovered hub mRNAs on apoptosis and angiogenesis are demonstrated in Figure 1.

#### 2.2.1 Methylated RNA Immunoprecipitation Sequencing

Total RNA was isolated and purified using TRIzol reagent (Invitrogen, Carlsbad, CA) following the manufacturer’s procedure. The RNA amount and purity of each sample was quantified using NanoDrop ND-1000 (NanoDrop, Wilmington, DE). The RNA integrity was assessed by Bioanalyzer 2100 (Agilent, Santa Clara, CA) with RIN number >7.0 and confirmed by electrophoresis with denaturing agarose gel. Afterward, Epicentre Ribo-Zero Gold Kit (Illumina, San Diego, CA) was used to deplete ribosomal RNA (rRNA) from total RNA. The ribosomal-depleted RNA was fragmented into small pieces using Magnesium RNA Fragmentation Module (New England Biolabs, Ipswich, MA). Then the cleaved RNA fragments were incubated with m^6^A-specific antibody (Synaptic Systems, Walldorf, BW, DE) in immunoprecipitation (IP) buffer (50 mM Tris-HCl, 750 mM NaCl, and 0.5% Igepal CA-630). Subsequently, the IP RNA was reverse transcribed to create cDNA by SuperScript™ II Reverse Transcriptase (Invitrogen, Waltham, CA). Eluted fragments containing m^6^A and untreated input control fragments were converted to construct the strand-specific cDNA library by dUTP method (Dominissini et al., 2013). The average insert size of the final cDNA library was 300 ± 50 bp. We finally performed 2 × 150 bp paired-end sequencing (PE150) with an illumina Novaseq™ 6000 (LC-Bio Technology CO., Ltd., Hangzhou, China) (Meng et al., 2014; Li M. et al., 2019).

#### 2.2.2 RNA-Seq

Total RNA was isolated and purified using TRIzol reagent (Invitrogen, Carlsbad, CA). For RNA-seq analysis, rRNA was depleted, according to the protocol of the Epicentre Ribo-Zero Gold Kit (Illumina, San Diego, CA). Subsequently, the ribosomal-depleted RNA was fragmented into small pieces using Magnesium RNA Fragmentation Module (New England Biolabs, Ipswich, MA). The fragments were converted to construct the strand-specific cDNA library with dUTP method and were sequenced by illumina Novaseq™ 6000 (LC-Bio Technology CO., Ltd., Hangzhou, China) (Wang Q. et al., 2020).

#### 2.2.3 Bioinformatic Analysis

Fastp software was used to remove the reads containing adaptor contamination, low quality bases, and undetermined bases with default parameters (Chen et al., 2018). Then sequence quality of IP and input samples were verified with fastp. HISAT2 were used to map reads to the reference genomeRattus norvegicus (Version 101) (Kim et al., 2015). Mapped reads of IP and input libraries were then provided with “exomePeak” package (Meng et al., 2014). m^6^A peaks from the corresponding libraries were visualized with IGV software (Broad Institute, Cambridge, MA) (Thorvaldsdóttir et al., 2013). MEME and HOMER software were used for *de novo* and known motif findings followed by localization of the motif with respect to peak summit (Bailey et al., 2009). The information of m^6^A peaks was obtained by intersection with gene architecture using “ChIPseeker” package (Yu et al., 2015). Then StringTie was used to obtain expression for all mRNAs from input libraries by calculating Fragments Per Kilobase of exon model per Million mapped fragments (FPKM) (Pertea et al., 2015). The differentially expressed mRNAs were selected with |log2FC|>1 and *p* value < 0.05 by “edgeR” package (Robinson et al., 2010).

### 2.3 Validation

Expression of top 10 hypermethylated and downregulated mRNAs was first validated with qRT-PCR testing. Subsequently, the m^6^A RNA methylation level was measured in histological and cellular level. qRT-PCR and Western blot were performed to verify the expression of genes and corresponding proteins. MeRIP-quantitative real-time PCR was performed to measure m^6^A methylation level of hub genes. Flow cytometry and Tunel assay were carried out to assess the apoptosis level. Cell counting kit-8 (CCK-8), 5-ethynyl-20-deoxyuridine (EdU), and tube formation assay were performed to test cell proliferation and tube formation ability.

#### 2.3.1 Quantification of Total m^6^A Methylation Level

m^6^A RNA methylation level was detected using the EpiQuik™ m^6^A RNA Methylation Quantification kit (Epigentek, New York, NY) according to the manufacturer’s protocol (Liu et al., 2020). Briefly, a negative control and a standard curve consisting of six different concentrations (ranged from 0.02 to 1 ng of m^6^A) were prepared. There was 200 ng of total RNA used for each reaction. After RNA binding to the 96-well plates, diluted capture anti-m^6^A antibodies were added, then 100 µl of developer solution to each well, and incubated at room temperature for 10 min without light. There was 100 µl of stop solution added afterward to each well to stop enzyme reaction. The optical density at 450 nm was measured using a microplate reader (Thermo Fisher Scientific, Waltham, CA). Percentage of m^6^A within the total RNA was calculated (Dang et al., 2020; Liu et al., 2020).

#### 2.3.2 Screening Strategy for Angiogenesis and Apoptosis Related Genes

We first obtained m^6^A methylation regulated hub genes by qRT-PCR, then, the hub genes were interacted with genes related to angiogenesis and apoptosis in the GeneCards. Results were visualized by Venn diagram (Liang et al., 2019).

#### 2.3.3 MeRIP-Quantitative Real-Time PCR

RNA sample from myocardial tissue and H9c2 cells was fragmented (300 nt) after incubation with fragmentation buffer at 94°C for 4 min. A total of 5% of fragmented RNA was saved as input control. The procedure of m^6^A-IP sample preparation was similar to that of MeRIP-seq (Deng et al., 2021). Finally, both input control and m^6^A-IP samples were subjected to qRT-PCR with gene-specific primers listed in Supplementary Table S1.

#### 2.3.4 qRT-PCR

Total RNA was extracted from cells and myocardial tissues and complementary DNA (cDNA) was synthesized using total RNA with the PrimeScript™ RT reagent kit with gDNA Eraser (TaKaRa, Kusatsu, Japan) according to the manufacturer’s instructions. qRT-PCR was performed with the Pro-17 Steponeplus system (Applied Biosystems, Carlsbad, CA) (Saeidi et al., 2018). Glyceraldehyde-3-phosphate dehydrogenase (GAPDH) was used as internal control to normalize the expression of genes. There are 25 pairs of primer listed in Supplementary Table S2. Relative expression of differentially expressed genes were analyzed with the 2^−△△CT^ method (Jozefczuk and Adjaye, 2011).

#### 2.3.5 Western Blot Analysis

H9c2 cells and HUVECs were lysed using RIPA lysis buffer (Beyotime, Shanghai, China). Protein concentration was determined using the BCA Protein Assay kit (New Cell and Molecular Biotech, Suzhou, China). Specifically, the corresponding protein was separated with 7.5 and 10% SDS-PAGE and transferred to polyvinylidene difluoride membranes. Afterward, the membranes were blocked with 5% skim milk for 2 h in room temperature (Qiu et al., 2020). They were incubated with primary antibodies against GAPDH (Proteintech, Wuhan, China); Tet1 (Signalway Antibody, College Park, MD); Hadh (Signalway Antibody, College Park, MD); Kcnn1 (Signalway Antibody, College Park, MD); Caspase3 (Proteintech, Wuhan, China); and Cleavd-caspase3 (Cell Signaling Technology, Danvers, MA); bcl-2 (Abcam, Cambridge, MA); Bax (Abcam, Cambridge, MA); HIF-1α (Abcam, Cambridge, MA); and Mettl3 (Proteintech, Wuhan, China) overnight at 4°C. Then, the membranes were incubated with horseradish peroxidase-conjugated secondary antibodies (Abcam, Cambridge, MA) for 1 h. The bands were analyzed with chemiluminescence Western blotting detection system (Tanon, Shanghai, China) (Gu et al., 2018; Ren et al., 2018; Li X. et al., 2020).

#### 2.3.6 Flow Cytometry

Annexin V-Fluorescein isothiocyanate (FITC) and propidium iodide (PI) staining kit (Vazyme, Nanjing, China) were used to identify apoptotic H9c2 cells. Flow cytometry analysis was performed through Cytoflex (Beckman Coulter, Brea, CA) and data was analyzed using FlowJo software (Tree Star, SanCarlos, CA). The second quadrant (FITC+/PI+) showed the late apoptotic cells and the fourth quadrant (FITC+/PI-) presented the early apoptotic cells (Luo et al., 2019; Zhang et al., 2019; Wei J. et al., 2020; Li Y. et al., 2020).

#### 2.3.7 Tunel Analysis

TUNEL BrightGreen Apoptosis Detection kit (Vazyme, Nanjing, China) was used for Tunel staining based on the manufacturer’s instructions. Briefly, the H9c2 was fixed in 4% PFA at 4°C for 25 min. Then the cells were incubated with Proteinase K (20 μg/ml) for 5 min. After incubation with 1×equilibration buffer for 30 min, the cells were treated with BrightGreen Labeling Mix and Recombinant TdT Enzyme for 1 h at 37°C. The nucleus was stained with DAPI (2 μg/ml) away from light for 10 min (Shi et al., 2021). The Tunel staining images were photographed with a fluorescence microscope (Bio-tek, Winusky, VT) (Zhang et al., 2016; Li T. et al., 2019; Luo et al., 2019; Sharma et al., 2021).

#### 2.3.8 Cell Counting Kit-8

Cell suspension with a concentration of 5×10^3^ each well was digested with trypsin and inoculated into a 96-well plate with 100 μL per well (Yu et al., 2021). After conventional culture for 24, 48, and 72 h, 10 μl CCK-8 solution (MedChemExpress, Shanghai, China) was added to each well and incubated for another 2 h. The absorbance value at 450 nm was finally measured by enzyme labeling instrument (Chen et al., 2019; Chen and Ling, 2019; Li X. et al., 2020; Zhou et al., 2020; Sun et al., 2021).

#### 2.3.9 EdU Assay

EdU incorporation assay kit (Ribobio, Guangzhou, China) was used for the measurement of cell proliferation (Liu et al., 2018). The cell suspension with a concentration of 8×10^3^ each well was digested with trypsin and inoculated into a 96-well plate with 100 μl per well, followed by the addition of 50 μM EdU diluent. After 2 h, cells were fixed in 4% paraformaldehyde, cultivated with 100 μl of 0.5% Triton X-100 and mixed with 100 μL of 1×Apollo^®^ 567 fluorescent staining solution (Xu et al., 2019). The cell nucleus was finally subjected to DAPI staining in a dark environment. Images were finally obtained from Cytation1 (Bio-tek, Winusky, VT).

#### 2.3.10 Tube Formation Assay

Matrigel (BD Biosciences, Franklin Lake, NJ) was used for tube formation assay to assess the tube-forming ability of HUVECs (Cheng et al., 2017). Briefly, it was dissolved at 4°C overnight, and each well of the 96-well plate was then coated with 50 μl of Matrigel. The plate was then left to polymerize at 37°C for 1 h incubation. The HUVECs (2×10^4^ cells/100 μl) were then seeded into each well. After 6 h of incubation at 37°C, tube formation was photographed at ×50 magnification. Tube formations were calculated with the number of branches using ImageJ software (National Institutes of Health, Bethesda, MD) (Li et al., 2017; Hanlon et al., 2019).

### 2.4 Statistical Analysis

Data were presented as mean with standard deviation. Results of qRT-PCT, MeRIP-qRT-PCR, total m^6^A methylation level, tube formation assay, and flow cytometry were analyzed with Student’s t-test. CCK-8 assay data across 24, 48, and 72 h was analyzed with two-way analysis of variance (ANOVA). Statistical significance was considered with *p* value less than 0.05. All analyses were performed using SPSS 22.0 (International Business Machines Corporation, Armonk, NY) and GraphPad Prism 9 (GraphPad, San Diego, CA).

## 3 Results

### 3.1 Overview of Methylated RNA Immunoprecipitation Sequencing

In the MeRIP-seq library, on average 86,616,150 and 90,985,883 valid reads were obtained in two groups of myocardial samples, while 81,704,807 and 90,279,353 valid reads were obtained in the RNA-seq library (Supplementary Table S3). In myocardial IP samples, the average mapping ratios of valid reads in the SO and the MI groups were 90.79 and 91.50%, respectively. The average mapping ratios of valid reads were 92.53 and 91.78% in the input samples (Supplementary Table S4). The valid data that mapped to the reference genome can be defined as the alignment to exon, intron, and intergenic according to the regional information. The average ratios of IP and input samples to exons were 67.91 and 55.20% in the SO group while 68.34 and 52.29% in the MI group, respectively (Supplementary Figure S1).

### 3.2 Mettl3 Induced Upregulation of m^6^A Methylation Level in Myocardial Tissue, H9c2 Cells, and HUVECs

**FIGURE 2:**
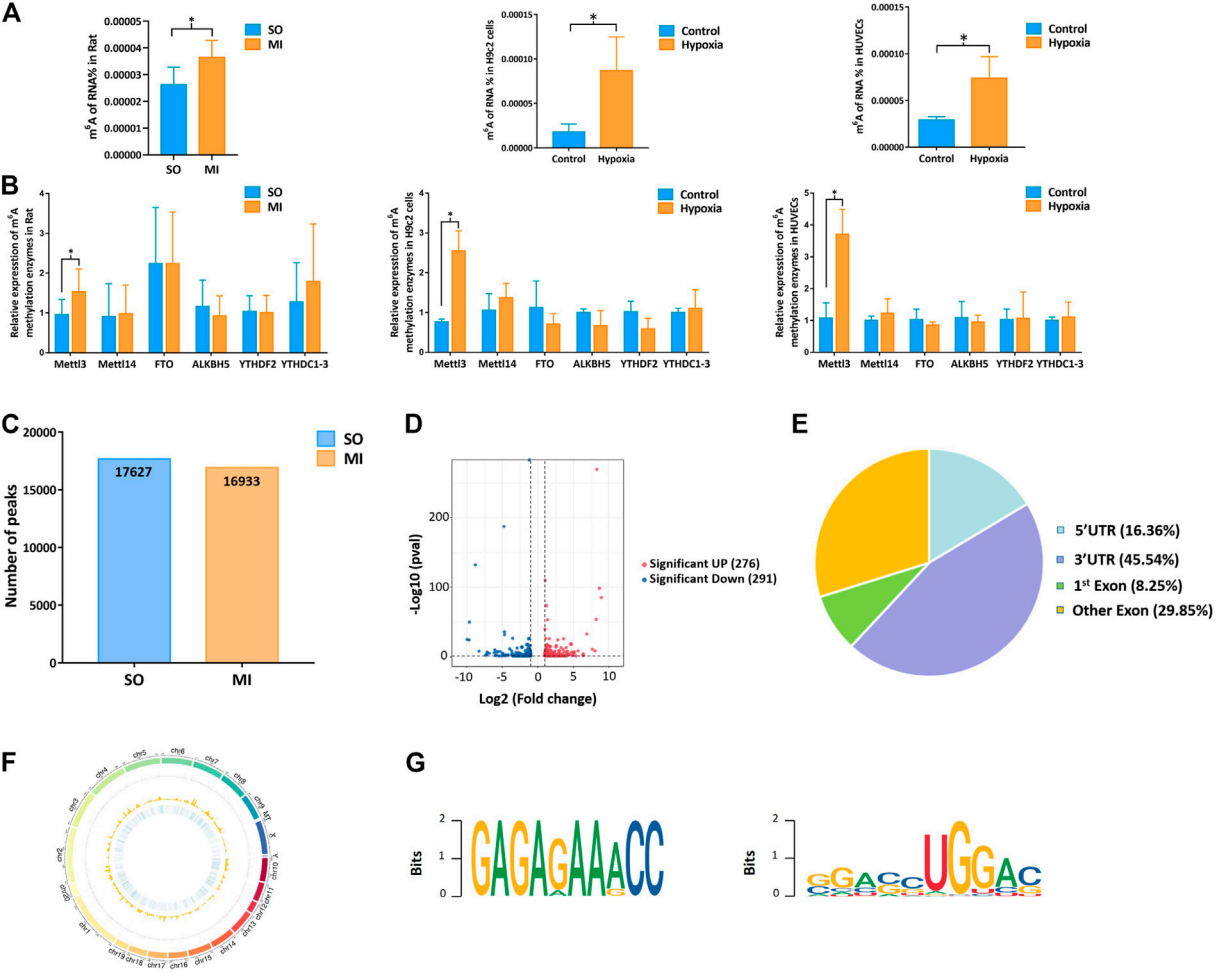
Mettl3 induced upregulation of m^6^A methylation level in myocardial tissue, H9c2 cells, and HUVECs. **(A)** m^6^A methylation level of MI rat and hypoxic cells; **(B)** expression of m^6^A methylation enzymes in myocardial tissue samples and cells by qRT-PCR; **(C)** number of peaks in the SO and MI group; **(D)** volcano plots for differentially methylated m^6^A peaks; **(E)** distribution of differentially methylated m^6^A peaks in the SO and MI group; **(F)** distribution patterns of differentially m^6^A methylated peaks on chromosomes; **(G)** sequence motif for the m^6^A peak regions. UTR, untranslated region; Exon, expressed region; SO, sham operated; MI, myocardial infarction. *p < 0.05.

Upregulation of Mettl3 was first observed *in vivo* (rat MI model) and *in vitro* (hypoxic H9c2 cells and HUVECs), followed by upregulation of m^6^A methylation level (Figures 2A,B). Through the analysis of MeRIP-seq, we identified 17,627 distinct m^6^A peaks in 9889 mRNAs in the SO samples and 16,933 distinct m^6^A peaks in 9764 mRNAs in the MI samples (Figure 2C). There were 567 significantly variated peaks identified, in which 276 peaks were upregulated and 291 were downregulated (Figure 2D). The top 20 differentially m^6^A methylated peaks are shown in Table 1. We analyzed the distribution patterns of differentially m^6^A methylated peaks. A total of 45.54% of the m^6^A methylated peaks harbored in the 3′UTR and 16.36% enriched in the 5′UTR (Figure 2E). All differentially m^6^A methylated peaks within mRNAs were mapped to chromosomes (Figure 2F). The top five chromosomes harboring the most m^6^A peaks were listed as follows: chr1 (646), chr10 (436), chr11 (130), chr12 (176), and chr13 (170). Finally, we conducted motif prediction for samples and demonstrated the predicted motif in Figure 2G.

**TABLE 1:** Top 20 differentially methylated mRNAs

mRNA	Chromosome	Peak region	Peak start	Peak end	*p*-value	Log2FC	Up/Down
Bach1	chr11	3′ UTR	27,397,948	27,398,514	6.30E-86	8.96	Up
Rrn3	chr10	3′ UTR	3,213,487	3,213,996	9.99E-270	8.32	Up
Senp5	chr11	Exon	72,064,886	72,067,150	2.45E-09	8.06	Up
LOC100910130	X	Exon	156,395,518	156,397,956	1.00E-11	7.68	Up
Hnrnph3	chr20	Exon	27,176,410	27,177,224	1.26E-33	6.91	Up
Gspt1	chr10	Exon	4,412,071	4,426,636	8.50E-03	6.39	Up
Bdkrb1	chr6	3′ UTR	129,439,859	129,440,455	1.20E-04	6.38	Up
Scart1	chr1	Exon	212,701,635	212,704,224	7.20E-02	5.70	Up
Natd1	chr10	5′ UTR	45,065,415	45,071,643	2.90E-02	5.39	Up
Gata6	chr18	Exon	2,417,213	2,417,483	6.46E-08	5.18	Up
Tap2	chr20	5′ UTR	3,995,544	3,996,581	7.94E-26	−9.94	Down
Nfat5	chr19	5′ UTR	38,533,016	38,533,316	3.98E-25	−9.69	Down
Sp3	chr3	Exon	59,646,075	59,646,253	1.25E-50	−9.63	Down
Cd248	chr1	Exon	220,353,416	220,354,637	1.26E-50	−8.79	Down
AABR07048992	chr5	5′ UTR	99,032,868	99,033,107	1.00E-132	−8.25	Down
Nfs1	chr3	5′ UTR	151,687,398	151,688,149	6.76E-09	−7.34	Down
Rpusd2	chr3	Exon	110,836,072	110,836,252	2.18E-04	−7.17	Down
Ranbp2	chr20	Exon	28,047,206	28,047,481	9.77E-06	−6.81	Down
Capns1	chr1	Exon	91,063,753	91,063,928	6.30E-11	−6.04	Down
Raver2	chr5	Exon	119,938,132	119,938,341	1.50E-02	−5.95	Down

FC, fold change.

### 3.3 Hub mRNAs Associated with Angiogenesis and Apoptosis

**FIGURE 3:**
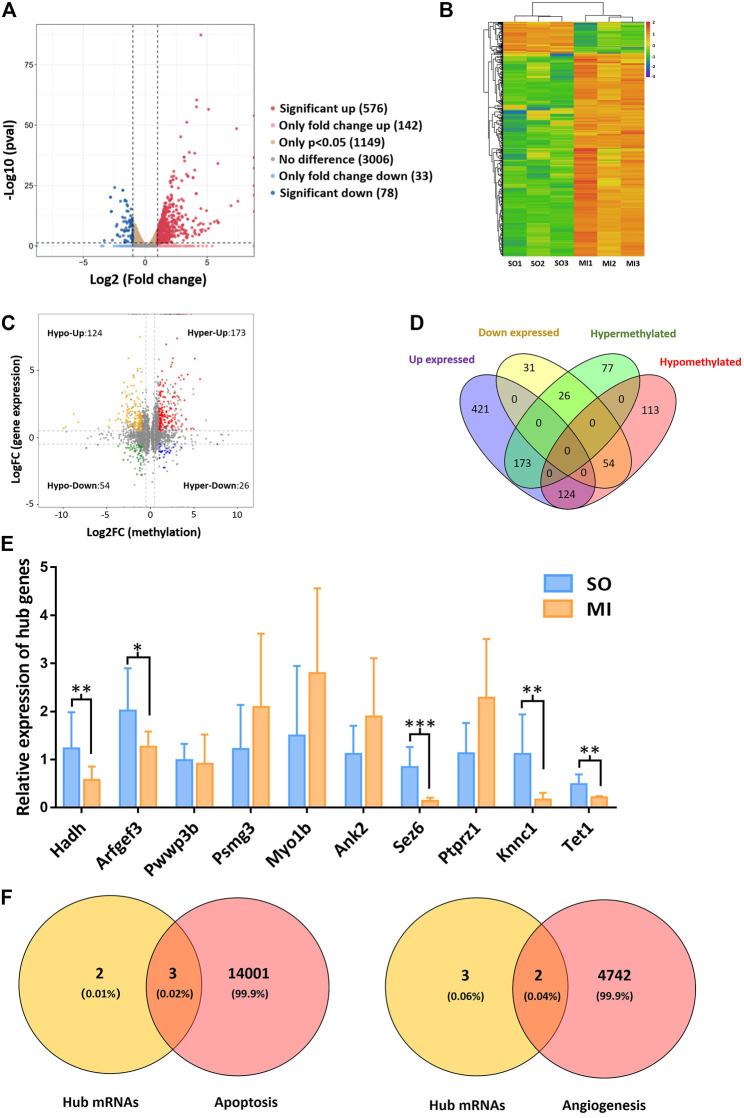
Hub mRNAs associated with angiogenesis and apoptosis. **(A)** Volcano plots for differentially expressed mRNAs; **(B)** heatmap plots for differentially expressed mRNAs; **(C)** four-quadrant graph for m^6^A methylation regulated mRNAs; **(D)** Venn diagram showing the relationships between m^6^A methylation and mRNAs expression; **(E)** validation of the top 10 hypermethylated and downregulated mRNAs in MI rats; **(F)** Venn diagram showing the relationship between hub mRNAs and apoptosis/angiogenesis. Hypo-Up, hypomethylated and upregulated; hyper-Up, hypermethylated and upregulated; Hypo-Down, hypomethylated and downregulated; hyper-Down, hypermethylated and downregulated. SO, sham operated; MI, myocardial infarction. *p < 0.05, **p < 0.01, ***p < 0.001.

Based on the results of RNA-seq, 576 mRNAs were significantly upregulated, and 78 mRNAs were downregulated. The top 20 differentially expressed mRNAs are listed in Table 2. Volcano plots and heatmap plots of differentially expressed mRNAs are shown in Figures 3A,B. Combined analysis of m^6^A methylation and mRNAs expression levels was performed according to the following thresholds: |log2FC|>1, *p* < 0.05 for m^6^A methylation and |log2FC|>0.5, *p* < 0.05 for mRNAs expression. As a result, we obtained 377 mRNAs where their m^6^A peaks and mRNA expression both changed significantly. The relationships between m^6^A methylation and mRNAs expression are shown in the four-quadrant graph and Venn diagram (Figures 3C,D). Accordingly, there were 124 significantly hypomethylated and upregulated mRNAs, 26 significantly hypermethylated and downregulated mRNAs, 173 significantly hypermethylated and upregulated, and 54 significantly hypomethylated and downregulated mRNAs. The top 10 hypermethylated and downregulated mRNAs are list in Table 3. Meanwhile, we provided the detailed information of the top 10 hypermethylated and upregulated mRNAs, the top 10 hypomethylated and downregulated mRNAs, and the top 10 hypomethylated and upregulated mRNAs in Supplementary Tables S5–S7. Expression of hub mRNAs, including Hadh, Arfgef3, Sez6, Psmg3, Kcnn1, Tet1, Myo1b, Ptprz1, Ank2, and Pwwp3b were compared between the SO and the MI groups by qRT-PCR testing. Among them, the expression of Hadh, Arfgef3, Sez6, Kcnn1, and Tet1 were significantly downregulated in the MI group (Figure 3E). After interaction of the above three hub mRNAs with apoptosis and angiogenesis-related genes in GeneCards, Hadh, Tet1, and Kcnn1 were finally identified to be significantly associated with apoptosis, and Tet1 and Kcnn1 were significantly correlated to angiogenesis (Figure 3F).

**TABLE 2:** Top 20 differentially expressed mRNAs.

mRNA	Chromosome	Gene start	Gene end	*p*-value	Log2FC	Up/Down
Gm47305	chr6	91,680,317	91,680,386	8.33E-67	12.06	Up
Ccn4	chr7	107,695,215	107,723,772	1.10E-23	8.30	Up
Mmp12	chr8	5,606,592	5,616,493	1.27E-15	8.28	Up
Col11a1	chr2	216,863,428	217,056,523	1.12E-57	7.95	Up
Grem1	chr3	105,203,309	105,214,989	1.22E-16	7.65	Up
Comp	chr16	20,798,437	20,807,070	1.57E-68	7.59	Up
Card14	chr10	108,440,950	108,468,310	3.02E-19	7.48	Up
Angptl7	chr5	165,312,130	165,316,652	2.84E-49	7.37	Up
Fam180a	chr4	62,844,971	62,860,446	4.73E-33	7.36	Up
Ccn5	chr3	160,207,913	160,219,331	1.24E-134	7.18	Up
Ptprh	chr1	72,810,545	72,859,161	1.54E-05	−6.83	Down
Fam111a	chr1	229,003,961	229,019,527	3.80E-07	−4.96	Down
Gp6	chr1	73,040,901	73,064,641	2.80E-03	−4.22	Down
7SK	chr17	90,799,609	90,799,914	2.00E-06	−3.90	Down
AABR07069186	chr8	10,985,553	10,988,077	4.10E-04	−3.47	Down
Gzmc	chr15	35,392,162	35,394,792	3.81E-04	−3.41	Down
Sec14l5	chr10	10,591,251	10,629,735	2.36E-13	−3.34	Down
Pdzk1	chr2	198,965,685	198,999,323	5.50E-03	−3.31	Down
Klk12	chr1	99,706,780	99,711,004	3.10E-02	−3.25	Down
Alas2	X	23,167,696	23,187,341	2.90E-04	−3.18	Down

FC, fold change.

**TABLE 3:** Top 10 hypermethylated and downregulated mRNAs.

mRNA	Chromosome	m^6^A regulation	Regulation	FPKM of MI input	FPKM of SO input
Hadh	chr2	Up	Down	87.12	249.88
Arfgef3	chr1	Up	Down	0.40	1.10
Pwwp3b	X	Up	Down	0.53	1.11
Sez6	chr10	Up	Down	0.35	0.71
Psmg3	chr12	Up	Down	1.28	2.62
Kcnn1	chr16	Up	Down	0.85	1.70
Tet1	X	Up	Down	0.29	0.58
Myo1b	chr9	Up	Down	12.53	21.41
Ptprz1	chr4	Up	Down	0.25	0.41
Ank2	chr2	Up	Down	11.74	18.99

FPKM, fragments per kilobase of exon model per million mapped fragments; MI, myocardial infarction; SO, sham operated.

### 3.4 m^6^A Methylation Influenced by Hypoxia in a Time- and Dose-Dependent Pattern

**FIGURE 4:**
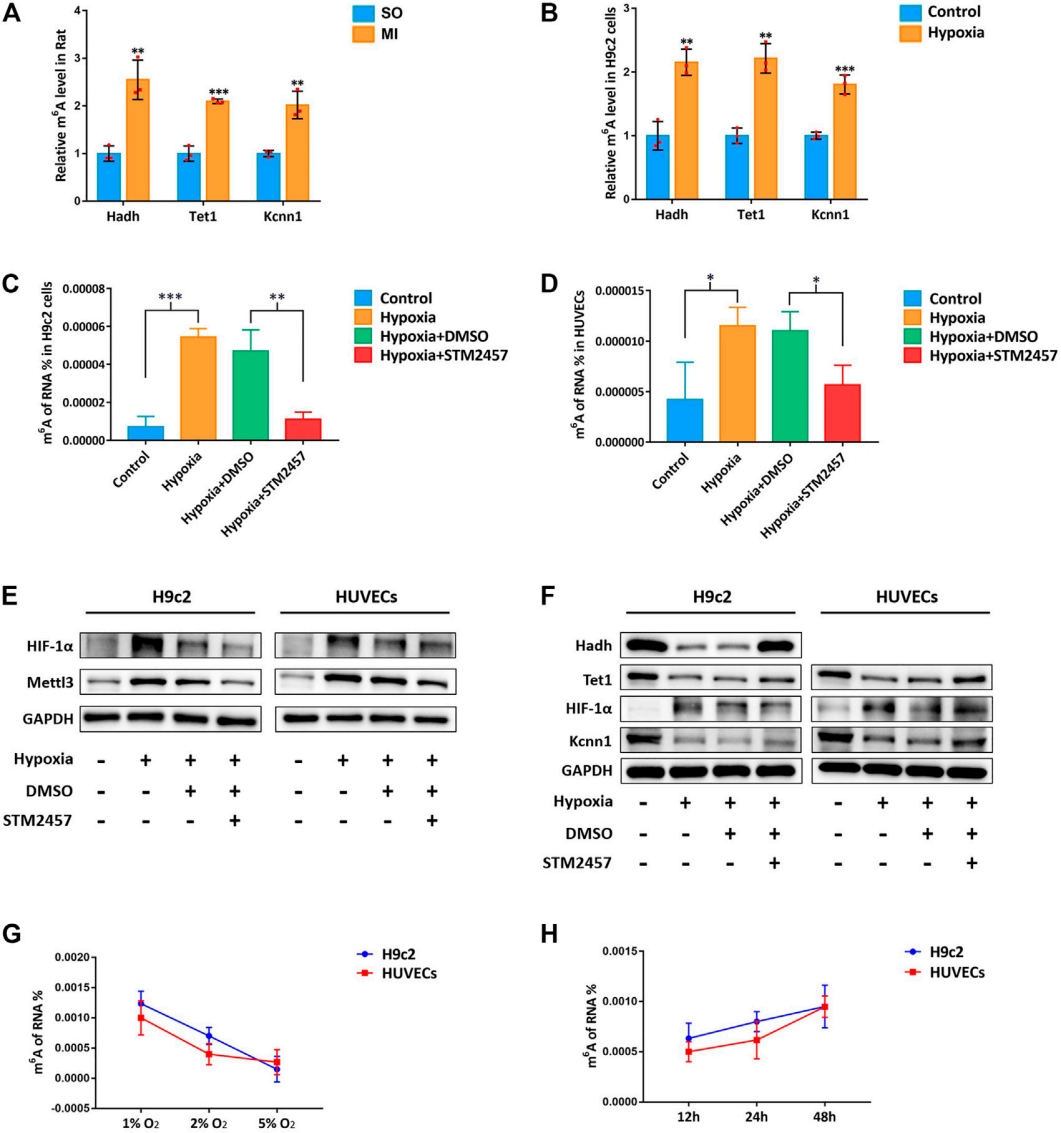
m^6^A methylation influenced by hypoxia in a time- and dose-dependent pattern. **(A)** MeRIP-qRT-PCR validation of hub mRNAs in MI rat; **(B)** MeRIP-qRT-PCR validation of hub mRNAs in H9c2 cells; **(C)** m^6^A methylation levels of H9c2 cells with Mettl3 inhibitor; **(D)** m^6^A methylation levels of HUVECs with Mettl3 inhibitor; **(E)** Mettl3 protein level of H9c2 cells and HUVECs with Mettl3 inhibitor. HIF-1α was used as positive control. GAPDH was used as loading control; **(F)** protein level of hub mRNAs in H9c2 cells and HUVECs with Mettl3 inhibitor; **(G)** m^6^A methylation levels of H9c2 cells and HUVECs with different concentrations of oxygen; **(H)** m^6^A methylation levels of H9c2 cells and HUVECs with different hypoxic duration. SO, sham operated; MI, myocardial infarction; DMSO, dimethyl sulfoxide. *p < 0.05, **p < 0.01, ***p < 0.001. *STM2457 is the inhibitor of Mettl3.

Upon our previous observation, m^6^A methylation level was upregulated by Mettl3 *in vivo* and *in vitro* (Figures 2A,B). We afterward detected upregulated m^6^A methylation level of hub mRNAs with MeRIP-qRT-PCR, which further confirmed our results from transcriptome-wide MeRIP-seq analysis (Figures 4A,B). With the inhibition of Mettl3 in H9c2 cells and HUVECs, downregulated m^6^A methylation level of total RNA and upregulated expression of hub mRNAs were detected (Figures 4C–F). After treating with different oxygen concentration (1% O_2_, 2% O_2_, and 5% O_2_) for 24 h, m^6^A methylation level in H9c2 cells and HUVECs progressively decreased with the increase of oxygen concentration (Figure 4G). Afterward, these cells were treated with different hypoxic duration (12, 24, and 48 h) at 1% of oxygen. m^6^A methylation level in H9c2 cells and HUVECs gradually increased with prolonged hypoxic duration (Figure 4H). Variation of m^6^A methylation in the simulated hypoxic context demonstrated a time- and dose-dependent pattern.

### 3.5 m^6^A Methylation Regulated Hub mRNAs Play a Role on Apoptosis *in vitro*

**FIGURE 5:**
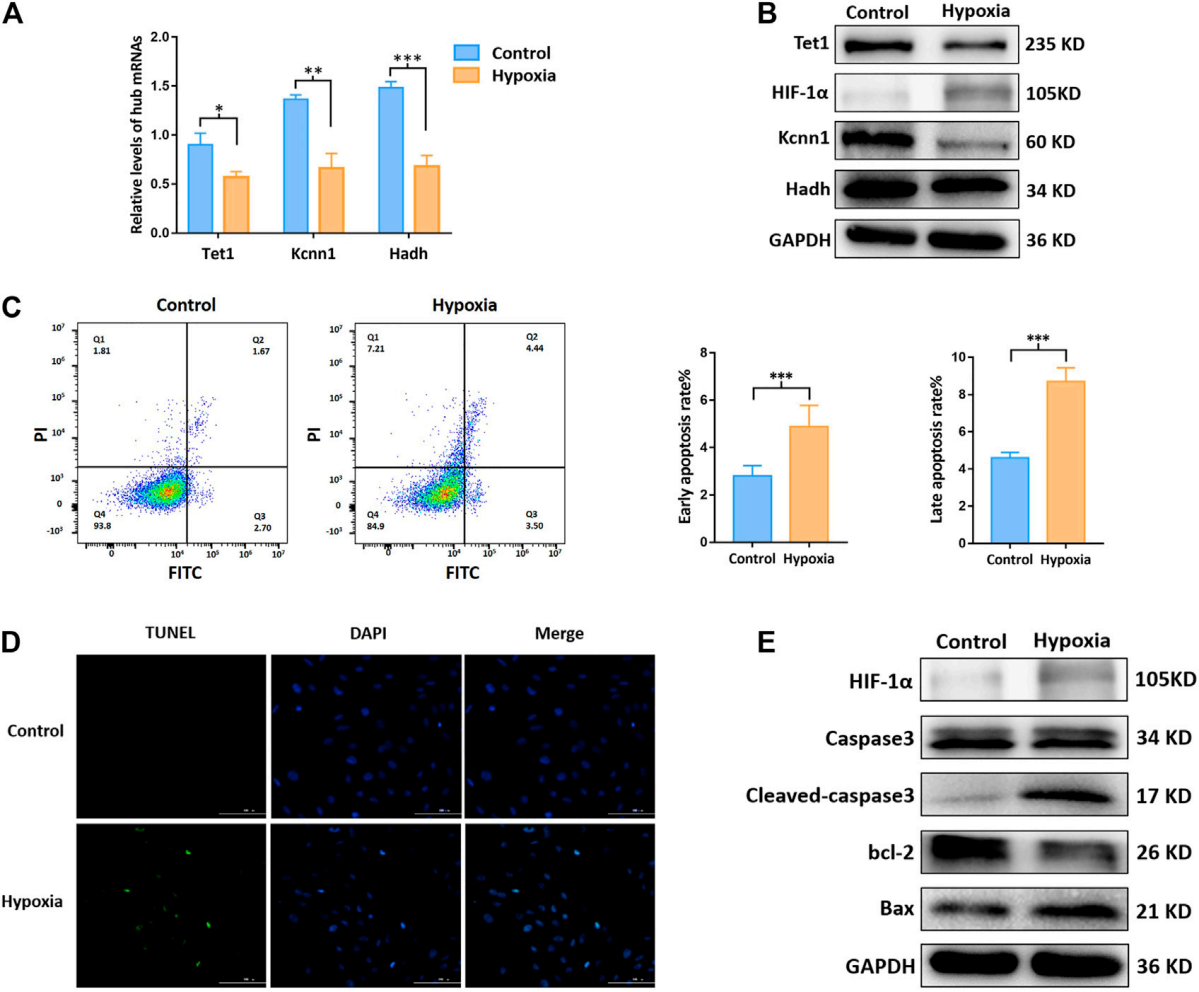
m^6^A methylation regulated hub mRNAs play a role on apoptosis *in vitro*. **(A)** Expression of apoptosis related mRNAs by qRT-PCR; **(B)** protein level of Tet1, Kcnn1, and Hadh by Western blot; **(C)** apoptosis rate by flow cytometry; **(D)** apoptotic status by Tunel staining; **(E)** protein level (Caspase3, cleaved-caspase3, bcl-2, Bax, and HIF-1α) by Western blot. *p < 0.05, **p < 0.01, ***p < 0.001.

As presented in Figures 5A,B, Hadh, Tet1, and Kcnn1 were significantly downregulated after hypoxia. The apoptosis rate of cardiomyocytes exposed to hypoxia increased significantly (Figures 5C,D). As compared to the control group, the expressions of cleaved-caspase3 and Bax increased significantly, while the expressions of bcl-2 decreased in the hypoxia group based on Western blot (Figure 5E).

### 3.6 m^6^A Methylation Regulated Hub mRNAs Play a Role on Angiogenesis *in vitro*

**FIGURE 6:**
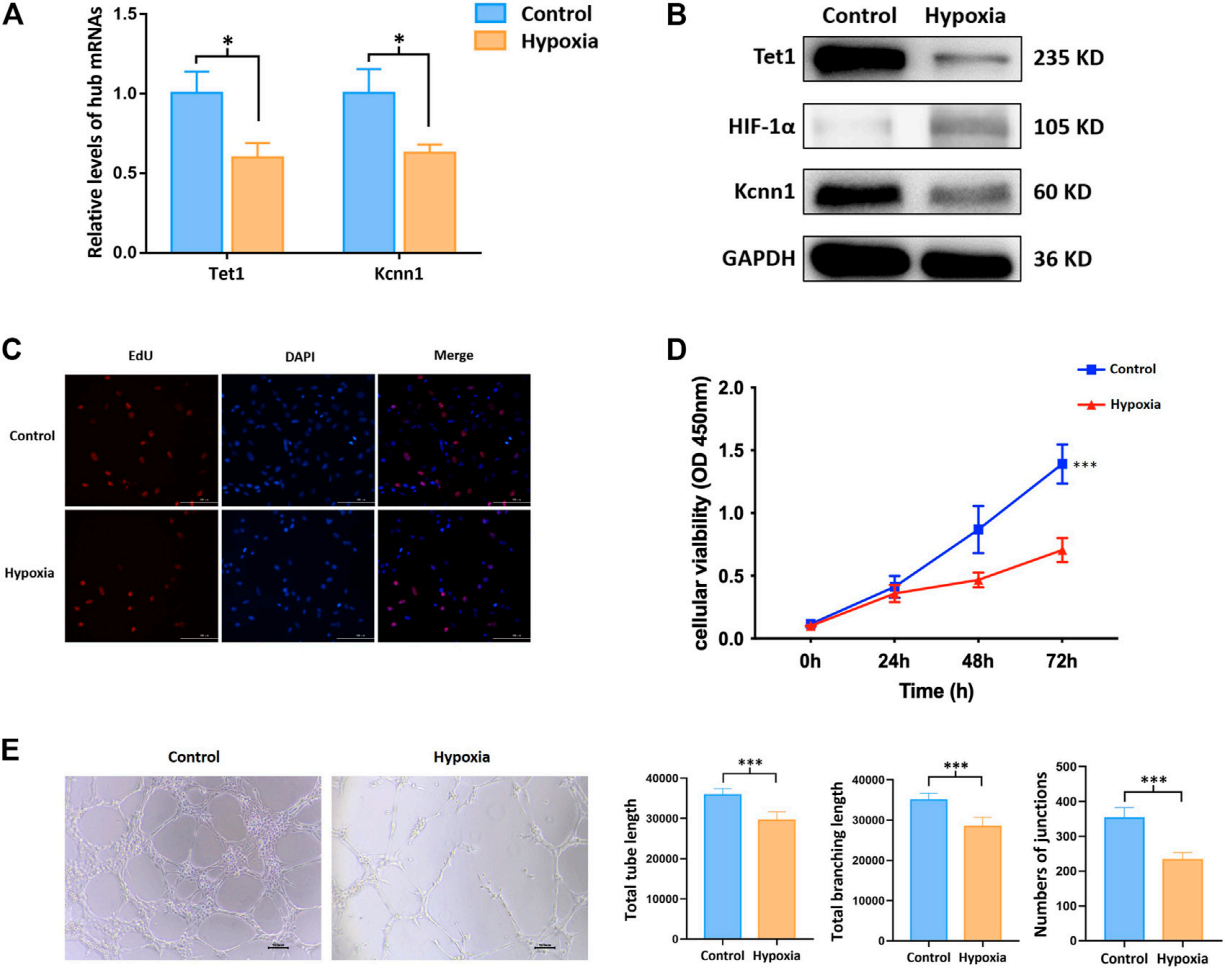
m^6^A methylation regulated hub mRNAs play a role on angiogenesis *in vitro*. **(A)** Expression of angiogenesis related mRNAs by qRT-PCR; **(B)** protein level of Tet1, Kcnn1, and HIF-1α by Western blot; **(C)** cell proliferation by EdU assay; **(D)** cell viability by CCK-8 assay; **(E)** tube formation. *p < 0.05; ***p < 0.001.

Kcnn1 and Tet1 determined by qRT-PCR and Western blot were significantly decreased in the hypoxia group (Figures 6A,B). The effects of hypoxia on cell proliferation and angiogenesis were revealed by CCK-8, EdU, and tube formation assay. Cell viability and EdU-positive cells were significantly decreased due to hypoxia (Figures 6C,D). All indicators of tube formation ability, including the total branching points, total tube length, and total loops, were markedly decreased in the hypoxia group (Figure 6E).

## 4 Discussion

DNA methylation and histone modification have been extensively investigated in genetic and cellular biology (Panneerdoss et al., 2018). However, roles of reversible RNA methylation in the cardiovascular field have been under development in recent years (Mongelli et al., 2020). In the hypoxia/reoxygenation-treated cardiomyocytes, low-expression of METTL3 and over-expression of ALKBH5 were observed to inhibit autophagy and apoptosis (Song et al., 2019). In addition, over-expression of ALKBH5 was verified to reduce infarct size, restore cardiac function, and facilitate cardiomyocyte proliferation after MI (Han et al., 2021). These promising results preliminarily revealed the roles of m^6^A methylation on mRNAs in ischemic heart diseases. However, information regarding systematic and comprehensive analysis is limited. In the current study, we observed the overall m^6^A methylation level was significantly upregulated with the upregulation of Mettl3 in MI rat and hypoxic cells. We demonstrated the landscape changes of m^6^A methylation on mRNA regulated apoptosis and angiogenesis in MI. Meanwhile, we explored the impact of oxygen concentration and hypoxic duration on m^6^A methylation. Upon which, several hypermethylated and downregulated mRNAs were successfully identified after MI by combined analysis of MeRIP-seq and RNA-seq. It further provided potential targets for treatment and scientific purposes. However, how and what performance of these m^6^A methylated mRNAs may have on MI need to be further elaborated.

Followed by the newly proposed mechanisms of upstream methylation on mRNA in this research field, the final destination is to compensate or improve the downstream functional impairment launched by MI. According to the literature review, apoptosis plays a role in the process of tissue damage after MI, which appears to have pathological and therapeutic implications (Krijnen et al., 2002). Therefore, the upcoming question in front of us was whether the m^6^A methylated mRNAs would play a role in the regulation of cardiomyocyte apoptosis. In this regard, we interacted our newly identified m^6^A methylated mRNAs and apoptosis related genes in the GeneCards and three of them were correlated to apoptosis (e.g., Tet1, Hadh, and Kcnn1). Over-expression of Tet1 increased cell apoptosis and inhibited cell growth in osteosarcoma cells (Teng et al., 2019). Over-expression of Hadh linked to cell apoptosis in acute myeloid leukemia (Wei L. et al., 2020). However, the relationship between Kcnn1 and apoptosis has not been validated according to previous studies. We afterward validated the m^6^A methylation regulated mRNAs on apoptosis in hypoxic H9c2 cells and found the m^6^A methylation level was upregulated in hypoxic H9c2 cells. Our results of MeRIP-qRT-PCR demonstrated that m^6^A methylation of three hub mRNAs was significantly upregulated, while their expression levels were downregulated in MI rat and hypoxic cells. In addition, inhibition of Mettl3 downregulated the overall m^6^A level and upregulated the expression of Hadh, Tet1, and Kcnn1 in hypoxic H9c2 cells. Apoptosis was accompanied with variations of hub proteins, for instance, decrease of bcl-2 and increase of Bax, Caspase3, and cleaved-caspase3. Taken together, these three hub mRNAs regulated by m^6^A methylation were preliminarily verified to impact the process of cardiomyocyte apoptosis after MI.

As the exacerbation of myocardial injury, it is likely leading to heart failure and sudden death (Zouggari et al., 2013). Early revascularization in the infarcted region is essential for promoting the survival of myocardial cells, reducing infarct size, and improving the prognosis of patients with MI (Keeley et al., 2003; Guo et al., 2017). Upon this perspective, angiogenesis has been reported to be an integral and indispensable part of the myocardial healing process following ischemic events (Cochain et al., 2013). The inhibition of angiogenesis accelerated heart failure in a murine model and various treatments were proposed to ameliorate infarction size, left ventricular remodeling, and cardiac function after MI via promoting angiogenesis in animal models of MI (Shiojima et al., 2005; Zeng et al., 2010; Shindo et al., 2016; Gou et al., 2020). In the past several decades, attentions have been shifted to investigate the mechanical associations between angiogenesis and epigenetics. mRNAs are responsible for protein translation and can directly regulate the synthesis of proteins such as vascular endothelial growth factor in the context of MI. However, the upstream regulation of mRNAs has not been well clarified. In the current study, two m^6^A methylation regulated mRNAs (e.g., Kcnn1 and Tet1) were detected by interaction analysis of five hub mRNAs and angiogenesis-related genes. Kcnn1 is one of the members in the calcium activated potassium channel subfamily. It was previously reported to be associated with the development of atrial fibrillation while its role in angiogenesis in the context of MI has not been reported (Park et al., 2014; Rahm et al., 2021). On the other hand, Tet1, as one of translocation enzymes mediating 5-methylcytosine (5mC) hydroxylation, was reported to participate in the facilitation of DNA demethylation (Gomes et al., 2020). In addition, inhibition of Tet1 expression may contribute to tumor growth and angiogenesis *in vivo* (Si et al., 2019). Upon this condition, we explored the impact of Kcnn1 and Tet1 on angiogenesis in hypoxia-induced HUVECs to further verify their roles after MI. Not surprisingly, in the context of hypermethylated and downregulated Kcnn1 and Tet1, CCK-8, EdU, and tube formation assay potentially indicated that hypoxia inhibits endothelial cell proliferation and angiogenesis. Meanwhile, we found the overall level of m^6^A methylation of total RNA was upregulated in hypoxic endothelial cells. Inhibition of Mettl3 downregulated the overall m^6^A level and upregulated the expression of Tet1 and Kcnn1 in hypoxic HUVECs. These results suggested that m^6^A methylated Kcnn1 and Tet1 may play an essential role in angiogenesis. Further studies to be conducted in knockdown and over-expression models would be helpful to validate the role of m^6^A methylation on mRNAs in the process of angiogenesis.

Similar to other MeRIP-seq and RNA-seq studies, the reproducibility of MeRIP-seq was considered poor. Fortunately, heart tissues of our animal model were consistently collected from similar regions in a standard environment, which guaranteed the homogeneity at the histological level to some extent. In addition, we performed a rescue experiment to verify the reproducibility of our sequencing results. However, it needs to be further verified with well-designed *in vitro* or *in vivo* studies. Finally, our results supplemented information of m^6^A methylated mRNAs in MI, which was conducive to the broadening of research ideas in this direction and provided reference for further studies.

## 5 Conclusion

The overview of upstream epigenetic changes after MI were demonstrated with emphasizing the essential role of m^6^A methylated mRNAs after MI. Five m^6^A methylation regulated mRNAs were newly identified by interaction analysis of MeRIP-seq and RNA-seq. In the context of m^6^A methylation, three of hub mRNAs were validated to impact the process of cardiomyocyte apoptosis and angiogenesis. Our study paved the way for future investigations aiming at exploring the upstream epigenetic mechanisms in the pathogenesis of MI.

## Data Availability

The data presented in the study are deposited in the GEO repository, accession number GSE189593.
