# Supplementary material for: Validated Impacts of N6-Methyladenosine Methylated mRNAs on Apoptosis and Angiogenesis in Myocardial Infarction Based on MeRIP-Seq Analysis
Source: Front Mol Biosci. 2022 Jan 28;8:789923. doi: 10.3389/fmolb.2021.789923 (PMC8831860; doi:10.3389/fmolb.2021.789923)

**Supplementary Materials**

**List of content**

**Table S1. Primer sequences of RNAs for MeRIP-qRT-PCR**

**Table S2. Primer sequences of RNAs for qRT-PCR**

**Table S3. Summary of reads quality control**

**Table S4. Summary of reads mapping to the rat reference genome**

**Table S5. Top 10 hypermethylated and upregulated mRNAs**

**Table S6. Top 10 hypomethylated and downregulated mRNAs**

**Table S7. Top 10 hypomethylated and upregulated mRNAs**

**Figure S1. Refer to the genome to compare the regional distribution**

**Table S1. Primer sequences of RNAs for MeRIP-qRT-PCR**

| **Gene Name** | **Species** | **Sequence** |
| --- | --- | --- |
| Kcnn1 | Rat | F:5' GTTCGGGATTGTCGTCATGG 3'  R:5' AGGAACAGCTGGATCTCTCG 3' |
| Tet1 | Rat | F:5' ACATTGCTGGAGACTGTCGA 3'  R:5' TTCTGTCACGGCCATCTTCT 3' |
| Hadh | Rat | F:5' CTGCCGATGAGTTTGTGGAG 3'  R:5' CCACAAGCTTCATCATGGGG 3' |

**Table S2. Primer sequences of RNAs for qRT-PCR**

| **Gene Name** | **Species** | **Sequence** |
| --- | --- | --- |
| GAPDH | Rat | F:5' GCATCTTCTTGTGCAGTGCC 3'  R:5' ACCAGCTTCCCATTCTCAGC 3' |
| Hadh | Rat | F:5' CTGCACTGTTGTTTCCAGGC 3'  R:5' GACCTGAGTGAATGCGGCTA 3' |
| Arfgef3 | Rat | F:5' CTCTATGCCCGCGGATCTC 3'  R:5' TGATGAATCACGCCCGCTC 3' |
| Sez6 | Rat | F:5' TTCCTTTGCAGGAGACGAGAG 3' |
|  |  | R:5' GCACCGCCAAATCCAAAGAA 3' |
| Psmg3 | Rat | F:5' GAAGCTCGACAGGCACATTG 3' |
|  |  | R:5' TTGGGACCAAAACCCTCAGC 3' |
| Kcnn1 | Rat | F:5' GCTGAGAAACACGTGCACAA 3' |
|  |  | R:5' TGGCCTGAAGGAACTTACGC 3' |
| Myo1b | Rat | F:5' GAGGCGAAGCAGGAGAAAGT 3' |
|  |  | R:5' GCTCGAAGCTGTTGTCCTCA 3' |
| Ptprz1 | Rat | F:5' GGCAAGGCACCTCAGATAGC 3' |
|  |  | R:5' ATCAGCACATCTCGTTCTATCCC 3' |
| Ank2 | Rat | F:5' CCGAGCTTCTTATCCCCACC 3'  R:5' CCCAAGCCCACCTACCTTC 3' |
| Pwwp3b | Rat | F:5' TTCCCTCTCCGACGAGAAGT 3'  R:5' TCACAGACATGATCGCCGAG 3' |
| Tet1  FTO  Mettl3  Mettl14  ALBKH5  YTHDF2  YTHDC1-3  FTO  Mettl3  Mettl14  ALBKH5  YTHDF2  YTHDC1-3  Tet1  Kcnn1 | Rat  Rat  Rat  Rat  Rat  Rat  Rat  Human  Human  Human  Human  Human  Human  Human  Human | F:5' GGTCTGTACTGGGAGACGC 3'  R:5' GTAGAGGTGCATGGCTACGG 3'  F:5' AGAATGTCTGTGACGATGTGG 3'  R:5' GCACTTTCTGTATCGATTGCC 3'  F:5' CACAGACCAGGCTAGAATTGC 3'  R:5' TTGCTCTTCCAGAGGACTCG 3'  F:5' TGAAGCGAAGCACAGATGGG 3'  R:5' GATAGTGCTGTCCCTCCCGA 3'  F:5' GCTTCGGTTGCAAGTTCCAG 3'  R:5' GGACCCGGCTGGATAGTTTT 3'  F:5' CAACAGACACAGCCATTGCC 3'  R:5' TAGATCCAGAACCCGCCTGA 3'  F:5' CGATTTTCAGGAGTTCGCCG 3'  R:5' GAGCAATACAAACCATTCCTTGC 3'  F:5' CGAGAGCGCGAAGCTAAGA 3'  R:5' GCTGCCACTGCTGATAGAAT 3'  F:5' GACACGTGGAGCTCTATCCAG 3'  R:5' AAGGTGGAGAGGGAGTACCA 3'  F:5' GAACACTGCCTCATGGGGAT 3'  R:5' TGTTAACTGAGGTCCTACCTGG 3'  F:5' CCGGTTGGAAACAAAGTCCC 3'  R:5' GCAAGCCAAGGCTCCTAAAC 3'  F:5' CCTTAGGTGGAGCCATGATTG 3'  R:5' TCTGTGCTACCCAACTTCAGT 3'  F:5' GAACAACCTCCACACCATCCT 3'  R:5' TAGTTCCCTCACACCTTTGCC 3'  F:5' CAGAACCTAAACCACCCGTG 3'  R:5' TGCTTCGTAGCGCCATTGTAA 3'  F:5' TCGGGGAAACCCTCAAATGTG 3'  R:5' CCATGACGACGATGCCAAAC 3' |

**Table S3.** **Summary of reads quality control**

| **Sample** | **Raw_Reads** | **Valid_Reads** | **Valid%** | **Q20%** | **Q30%** | **GC%** |
| --- | --- | --- | --- | --- | --- | --- |
| Control1_IP | 85613328 | 83673444 | 89.29 | 97.94 | 94.13 | 48.68 |
| Control2_IP | 90059320 | 87966524 | 89.04 | 98.12 | 94.59 | 48.79 |
| Control3_IP | 90378654 | 88208482 | 89.16 | 97.95 | 94.23 | 48.79 |
| MI1_IP | 96389326 | 94109178 | 89.28 | 97.98 | 94.30 | 49.33 |
| MI2_IP | 92010960 | 90037296 | 89.60 | 97.97 | 94.22 | 49.05 |
| MI3_IP | 90656054 | 88811176 | 90.05 | 98.11 | 94.54 | 48.92 |
| Control1_input | 76309778 | 74963578 | 88.55 | 98.12 | 94.41 | 47.06 |
| Control2_input | 92128904 | 90500744 | 88.39 | 98.20 | 94.59 | 47.02 |
| Control3_input | 80943904 | 79650098 | 88.87 | 98.18 | 94.54 | 46.98 |
| MI1_input | 87683028 | 86221232 | 88.71 | 98.17 | 94.53 | 47.40 |
| MI2_input | 93110634 | 91257024 | 88.24 | 98.14 | 94.48 | 47.43 |
| MI3_input | 95101328 | 93359802 | 89.01 | 98.18 | 94.58 | 46.72 |

**Table S4.** **Summary of reads mapping to the rat reference genome**

| **Sample** | **Valid reads** | **Mapped reads** | **Unique Mapped reads** | **Multi Mapped reads** |
| --- | --- | --- | --- | --- |
| Control1_IP | 78892638 | 71274457  (90.34%) | 59586958  (75.53%) | 11687499  (14.81%) |
| Control2_IP | 82685038 | 75531179  (91.35%) | 63563447  (76.87%) | 11967732  (14.47%) |
| Control3_IP | 83012256 | 75280801  (90.69%) | 63103743  (76.02%) | 12177058  (14.67%) |
| MI1_IP | 91357366 | 83038165  (90.89%) | 69481051  (76.05%) | 13557114  (14.84%) |
| MI2_IP | 86829384 | 79329134  (91.36%) | 66463789  (76.55%) | 12865345  (14.82%) |
| MI3_IP | 85841654 | 79228601  (92.30%) | 66703658  (77.71%) | 12524943  (14.59%) |
| Control1_input | 71051878 | 65695331  (92.46%) | 54309286  (76.44%) | 11386045  (16.02%) |
| Control2_input | 85497216 | 79229440  (92.67%) | 65771553  (76.93%) | 13457887  (15.74%) |
| Control3_input | 75363238 | 69679791  (92.46%) | 57901879  (76.83%) | 11777912  (15.63%) |
| MI1_input | 83809858 | 77081124  (91.97%) | 63283187  (75.51%) | 13797937  (16.46%) |
| MI2_input | 87776484 | 80315140  (91.50%) | 65946331  (75.13%) | 14368809  (16.37%) |
| MI3_input | 90019092 | 82711003  (91.88%) | 68410981  (76.00%) | 14300022  (15.89%) |

**Table S5** **Top 10 hypermethylated and upregulated mRNAs**

| **mRNA** | **Chromosome** | **m^6^A Regulation** | **Regulation** | **FPKM of MI Input** | **FPKM of SO Input** |
| --- | --- | --- | --- | --- | --- |
| Angptl7 | chr5 | Up | Up | 16.85 | 0.10 |
| LOC103689965 | chr20 | Up | Up | 93.31 | 1.60 |
| Grin3a | chr5 | Up | Up | 0.75 | 0.02 |
| Edar | chr20 | Up | Up | 0.63 | 0.02 |
| Olfm2 | chr8 | Up | Up | 0.60 | 0.03 |
| Scart1 | chr1 | Up | Up | 0.31 | 0.02 |
| Lrrtm3 | chr20 | Up | Up | 0.20 | 0.02 |
| Has1 | chr1 | Up | Up | 2.32 | 0.29 |
| Arntl | chr1 | Up | Up | 5.56 | 0.87 |
| Htr2b | chr9 | Up | Up | 0.69 | 0.13 |

**Table S6 Top 10 hypomethylated and downregulated mRNAs**

| **mRNA** | **Chromosome** | **m^6^A Regulation** | **Regulation** | **FPKM of MI Input** | **FPKM of SO Input** |
| --- | --- | --- | --- | --- | --- |
| Ciart | chr2 | Down | Down | 1.16 | 8.07 |
| Myo16 | chr16 | Down | Down | 0.32 | 2.15 |
| Ddn | chr7 | Down | Down | 0.88 | 4.87 |
| Rims1 | chr9 | Down | Down | 0.29 | 1.41 |
| Cacng4 | chr10 | Down | Down | 0.31 | 1.19 |
| Ubxn10 | chr5 | Down | Down | 0.27 | 0.91 |
| Cdh23 | chr20 | Down | Down | 1.48 | 4.33 |
| Paqr9 | chr8 | Down | Down | 2.91 | 7.69 |
| Mapk11 | chr7 | Down | Down | 0.51 | 1.31 |
| Papln | chr6 | Down | Down | 2.86 | 6.94 |

**Table S7 Top 10 hypomethylated and upregulated mRNAs**

| **mRNA** | **Chromosome** | **m^6^A Regulation** | **Regulation** | **FPKM of MI Input** | **FPKM of SO Input** |
| --- | --- | --- | --- | --- | --- |
| Prss35 | chr8 | Down | Up | 1.36 | 0.02 |
| Col9a2 | chr5 | Down | Up | 0.34 | 0.01 |
| Cpz | chr14 | Down | Up | 4.91 | 0.22 |
| Ccn2 | chr1 | Down | Up | 160.25 | 9.00 |
| C6 | chr2 | Down | Up | 5.55 | 0.33 |
| Klhl29 | chr6 | Down | Up | 1.47 | 0.10 |
| Piezo2 | chr18 | Down | Up | 5.57 | 0.51 |
| Tnfrsf8 | chr5 | Down | Up | 0.24 | 0.02 |
| Slc25a43 | X | Down | Up | 0.93 | 0.11 |
| Reep2 | chr18 | Down | Up | 0.50 | 0.06 |

**Figure S1. Refer to the genome to compare the regional distribution**


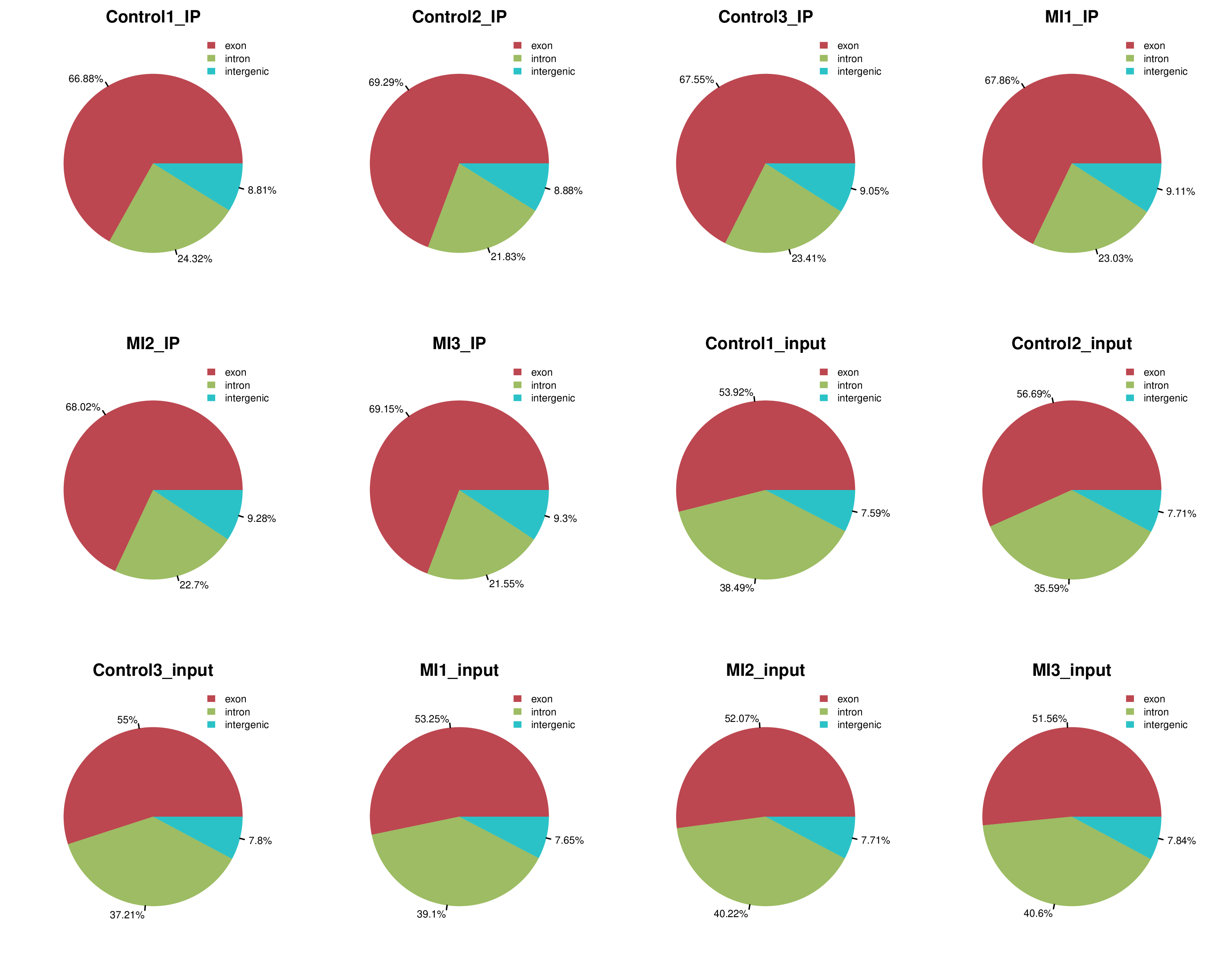

Supplement: Supplementary file 1 [file DataSheet1.docx]
